# Factors that Influence the Decision to Seek Help in a Police Population

**DOI:** 10.3390/ijerph17186891

**Published:** 2020-09-21

**Authors:** Carolyn Burns, Marla Buchanan

**Affiliations:** 1 Department of Educational and Counselling Psychology and Special Education, The University of British Columbia, Vancouver, BC V6T 1Z4, Canada; marla.buchanan@ubc.ca; 2 Burns Psychological Services, Langley, BC V2Y 0E2, Canada

**Keywords:** prevention, police mental health, police help seeking, organizational stress, resilience

## Abstract

Police officers face many competing pressures and demands. Exposure to potentially traumatic incidents and significant job-related stressors can place many at higher risk of developing physical and mental health problems. The police culture exerts a pronounced influence on officers, preventing some from asking for or receiving assistance. The stigma of being perceived as weak or incompetent, concerns about being labelled unfit for duty, and worry that accessing psychological support will impact future career advancement can affect the decision to seek help. The Enhanced Critical Incident Technique was utilized to investigate the following research question: What helps or hinders the decision to access psychological services in a police population? Qualitative interviews were conducted with 20 serving Royal Canadian Mounted Police officers in the lower mainland of British Columbia, Canada. The findings encompass five main themes: the importance of systemic factors, access to information and education, quality and influence of relationships, individual characteristics, and organizational processes that will increase the likelihood of accessing mental health services. The results contribute to the empirical literature by enhancing what is known about elements that influence an officers’ decision to seek psychological services, and factors that can enable officers to overcome barriers.

## 1. Introduction

### 1.1. The Culture of Policing

In general, “police culture” has been criticized as “overtly masculine, politically conservative, communally isolated, cynical, action-oriented, and marked by extreme loyalty among officers” [1] (p. 282). Empirical literature has consistently found that a majority of officers enter the policing profession to make a difference [2,3]. Specific training processes are utilized to initiate new officers into policing culture and promote behaviours designed to emulate an idealistic persona of strength, courage and control—characteristics of an “ideal” police officer [3,4,5]. Loyalty and commitment to the organization, especially to other officers, is effectively instilled through training and assimilation into the policing culture. Veteran officers are instrumental in training, but have been viewed as impediments to change if the workplace alterations are perceived as inconsistent with the established beliefs, values and traditions in a largely male-dominated culture [3,5,6]. It is particularly detrimental to efforts designed to alter the perception of fear and stigma often associated with mental illness or interest in accessing psychological assistance. Strong allegiance to the police culture and to colleagues can give rise to an “us/them” perception and potential isolation from family and friends [7]. The need to belong and be accepted can be critical to officer safety [8]. The paramilitary structure promotes conformity and unit integrity. Lack of conformity can lead to stigmatization and ostracization from the group that often becomes the officers’ primary support system [6,7,8]. As characteristics of the “ideal” officer include mental toughness, those indicating a need for psychological interventions may fear being ostracized from their group, the very group that they rely upon for their safety.

The Prime Minister of Canada authorized the Minister of Public Safety and Emergency Preparedness and the Minister of Health to design a National Action Plan for Canadian Public Safety Personnel who have been diagnosed with posttraumatic stress disorder [9] (p. 55). In 2016, R. Oliphant chaired the Standing Committee on Public Safety and National Security to develop a national strategy for occupational stress injuries among public safety personnel (PSP) [10]. In 2017, the National Defence and Canadian Armed Forces created a mental health training program, entitled Road to Mental Readiness (R2MR), that has now been rolled out to public service personnel through the Mental Health Commission of Canada [11]. The R2MR has been employed in various training programs for police personnel; however, there are few published studies [9,12,13] that have evaluated its effectiveness with this population. In a large-scale study of public safety personnel, Carleton et al. (2020) found “small but significant (ds < 0.20; ps > 0.05) changes in mental health symptoms, resilience, and work engagement, and a small but significant (*d* = 0.287; *p* < 0.05) reduction in stigma at post-training” [9] (p. 57). Szeto, Dobson and Knaak [12] used a meta-analytic method to investigate the effects of R2MR on resilience improvement and stigma reduction among first responders. They reported that among police service participants, the item “I plan to seek help for my mental health problems when needed” was greatly improved from baseline. They also reported that “6.3% of participants claimed that the program led them to seek professional help for their own mental health and/or enabled them to get a friend or colleague to seek professional help for their mental health. Overall, participants reported fewer stigmatizing attitudes towards those with mental health illnesses and felt more prepared to handle stressful and traumatic events in their workplace.” [12] (p. 265).

In a qualitative study on implementing cultural change within police organizations, Knaak et al. (2019) [13] found four main themes: (1) successful cultural uptake of the R2MR; (2) organizational factors contributing to successful cultural uptake that included cultural readiness, strong support from leadership, implementing R2MR as one piece of the puzzle; (3) implementation factors contributing to successful cultural uptake included ensuring positive group dynamics, and credibility of the trainers; (4) potential impacts of achieving successful cultural uptake included increased dialogue and the introduction of a common language, less judgement/more supportive culture, increased help-seeking and creating momentum [13] (pp. 325–365). Although there is limited research on the efficacy of R2MR within police populations, the preliminary findings are encouraging as a means to change policing culture [14].

In terms of research specifically with the Royal Canadian Mounted Police (RCMP), additional challenges are evident in that they are a national police force with a broad mandate and central command structure [15,16]. The action of a few can create a ripple effect, impacting the morale of officers and the perceptions of the public. As an example, in a study conducted by Perrott and Kelloway, the RCMP respondents reported experiencing a strong sense of peer support and meaning in their work, but lower supervisor support and morale than officers in other police agencies [17]. Overall, the cultural barriers that would impact help-seeking behaviours are present in the RCMP as in other police organizations [18]. As Carleton and others suggest [9] (p. 49), factors such as operational stressors, organizational stressors, familial stressors and individual differences must be taken into account when examining the mental health challenges of public safety personnel. As the authors cited here point out, there is a need for further research to inform our understanding of workplace interventions that will reduce stigma and increase help-seeking actions.

### 1.2. Risk Factors Within Police Populations

It has been well documented that police officers are exposed to potentially traumatic events at higher levels than the general population [19,20,21,22,23,24,25]. In a recent study, it was reported that 50.2% of RCMP and 36.7% of municipal/provincial police screened positively for one or more mental health disorders [9] (p. 509). Considerable evidence exists that the work of a police officer results in substantial early and ongoing exposure to potentially traumatic events [19,26,27,28].

Several authors identify early exposure to frequent potentially traumatic events within the first year on the job [29,30,31]. It is well known in the literature that police personnel experience higher rates of PTSD than the general public [10,32,33,34]. Research shows that the impact of exposure may lead to posttraumatic stress disorder (PTSD), depression, anxiety, relational stress, impaired social judgement and decreased work performance [4,33]. For example, Pitel, Papazoglou and Tuttle [22] explored the experiences of police officers during and after life-threatening incidents. Participants reported experiencing PTSD symptoms, depressive symptoms, sleep issues, problematic cognitive processing, lack of emotional regulation, and substance-misuse issues related to a potentially traumatic incident at work. Recently, Carleton and colleagues [9] found that Canadian public safety personnel screened positive for one or more mental health disorders, primarily PTSD (23.3%) or major depressive disorder (26.4%) (p. 38).

It is also well known that police work is stressful even without exposure to potentially traumatic incidents. According to Sherwood and colleagues [23] in the UK, research has suggested that as many as 90% of police personnel have experienced stress and poor mental health at work, and when compared to the general working population, police personnel are twice as likely to identify problems with work as the source of their mental health problems. The authors conducted a systematic review of the literature on the key risk factors for police mental health. In their review, between January 2008 and January 2018, they identified 20 research studies that described individual stressors, organizational stressors and operational stressors as key risk factors in police work. The individual factors they found were high levels of neuroticism, low levels of social support, and engagement in passive or avoidant coping strategies. Organizational risk factors found were high work demands, low resources, and low reward. The operational factors that put police officers at risk for mental health problems were related to the number of traumatic events, frequency between traumatic events as well as the specific type of traumatic incidents. In their review, the authors reported that police officers were susceptible to burnout that included both individual risk factors and organizational risk factors. “In addition to (lack of) social support in the workplace, frequently feeling overwhelmed, inflexible work hours, perceived unfairness, work overload, role ambiguity and a low level of work engagement were all associated with an increased risk of burnout” [23] (p. 697).

In a large Canadian study on prevalence rates of traumatic exposure among public safety personnel [33], RCMP participants reported the following: sudden violent death (95.7%), serious transportation accident (95.9%), physical assault (95.4%), sudden accidental death (95.1%), fire or explosion (88.4%), assault with a weapon (80.7%), sexual assault (80.7%) and severe human suffering (79.4%) to name the top 8 categories out of 16. According to the authors [34], “Public safety personnel who were RCMP (11.64), paramedics, (11.59) and municipal/provincial police (11.36) tended to report the highest mean levels of diverse exposure and the frequency of exposure ranged from 96.6% experiencing the event fewer than 6 times up to 89% experiencing the event 11+ times” (p. 42 and p. 48). In an earlier study by Carleton and colleagues [9], the authors found several demographic factors that contributed to the development of mental health problems: being unmarried, living in Western Canada, having less than a full university degree, having more years of service and being female were all risk factors (p. 3).

Korol and colleagues [35] assessed demographic and cognitive risk factors among Canadian police officers. The demographic risk factors were sex, education, marital status, and years of service whereas the cognitive risk factors examined were anxiety sensitivity and intolerance of uncertainty. The sample consisted of 979 police officers (708 men and 271 women) who completed measures on PTSD, panic disorder, social anxiety disorder, major depressive disorder, generalized anxiety disorder, intolerance of uncertainty and anxiety sensitivity. Only the variable of sex was significant for all symptom variables. Further, Intolerance for Uncertainty (IU)and Anxiety Sensitivity (AS) accounted for greater variance than sex on all mental disorder symptom measures (p. 14). The most recent study conducted by Angehrn, Krakauer and Carleton [32] also assessed IU and AS across PSP and found that there were significant differences across all PSP groups in terms of IU and AS who reported lower scores than clinical samples. The authors discussed how development of resiliency and strong coping strategies helped mitigate exposure to uncertain threat, a common risk factor among police personnel.

There is also evidence to suggest that personal interpretations of a traumatic event may be influenced by social contexts. Ricciardelli et al.’s [18] qualitative study points to the possibility of a “trauma hierarchy” among public safety personnel in their assessment of events that are more traumatizing than others and is based on how the trauma was experienced such as “(i) being first on the scene; (ii) responding to the scene, but not first on the scene; (iii) managing the scene; (iv) accumulated exposure to traumatic events over time, but without an ‘anchor event’; (v) indirect exposure to PTE, and (vi) unsupported and ‘old trauma ‘(e.g., trauma that is not recognized by PSP colleagues)” (p. 157). The authors point out that these findings may assist in the design of future interventions that address both stigma and helping-seeking initiatives.

### 1.3. Research Evidence on Police and Help-Seeking Behaviour

Research is growing on help seeking for mental health concerns among policing organizations. There is some discussion that the closed nature of the organization and distrust of outsiders have made it difficult for researchers to gain access to the population [7]. Despite the high risk of developing psychological difficulties from exposure to potentially traumatic events and extreme stress, police officers can be reticent to ask for help or receive professional mental health support [36]. In our search of the literature, five relevant studies were found on help seeking among police.

In the first study on structural factors potentiating mental health stigma, challenging awareness, and creating barriers to care among Canadian public safety personnel, Ricciardelli and colleagues [18] analyzed 828 open-ended final survey comments using a thematic analysis. Their study included 385 police (556 men and 269 women). The authors concluded that “system-level processes, underpinned with stigma directed toward persons with mental disorders, may be shaping care-seeking decisions, influencing how colleagues view care-seekers and reducing awareness of mental health needs among public safety personnel.” They discussed their findings under two main themes: (1) “Abusing the system”: Suspicion and cynicism and (2) systemic (institutional) stigma and perceptions of abuse. Participants voiced concerns about abusing the system and thus reducing the treatment and resources available to those who need mental health services. They stated that “particularly among police officers, participants reported experiencing system-level or structural stigma that they felt shaped the way mental disorders, specifically PTSD, were viewed within their organizations.” Mentally injured colleagues were perceived to be “playing the system” (p. 267). Police officers reported that they feared being discriminated against if they disclosed a mental health injury and worried they would receive negative evaluations on performance reviews. In the discussion, the authors summarized three help-seeking scenarios: (1) individuals need and seek help; (2) individuals do not need help, but still seek help, ultimately “playing the system”; and (3) individuals need, but do not seek help due to fears of stigmatization (p. 271).

A second study on police and help seeking examined male police officers and the stigma associated with seeking counselling [37]. Participants were 178 male law enforcement officers from a Southeast Wisconsin community, varying in age, policing experience, marital status, and cultural background. The officers completed questionnaires designed to measure gender role conflict (GRC), anticipated risks and benefits to seeking counselling, and perception of public and self-stigma. Results showed a connection between GRC and public perception and self-stigma. High levels of GRC resulted in increased rates of depression, anxiety, sexual aggression, use of maladaptive coping mechanisms, relationship difficulties and overall physiological distress. Lower scores on GRC correlated to higher self-esteem, marital satisfaction, emotional expressiveness and the likelihood of seeking psychological help. Officers who scored lower on GRC were more likely to see the benefits associated with counselling and perceived less public and self-stigma. The findings further highlighted the importance of educating officers on the benefits of counselling, and normalizing access to counselling in police populations.

In 2012, the Office of the Ombudsman for Ontario published a report about how the Ontario Provincial Police (OPP) dealt with Operational Stress Injuries (OSIs, Canadian Institute for Public Safety Research and Treatment (CIPSRT), 2019) [38]. Martin [39] conducted a comprehensive review involving nine team investigators, 191 interviews, including 81 complainants, 52 OPP staff, officers from the Provincial Police training academy, and a number of commanders and supervisors. An additional 51 interviews were conducted with external stakeholders including health services providers, psychologists, and psychiatrists. Martin’s [39] report highlighted the fact that regardless of a number of prevention and education strategies, stigma relating to psychological illness were still very much entrenched and pervasive within OPP ranks. Misconceptions about mental illness often lead those experiencing a psychological injury to feel “weak, inferior, damaged and sometimes dangerous (p. 80).” In a police culture where value is placed on strength and control, these beliefs may be greatly magnified. The overall membership appears to differentiate between those who are physically injured on the job requiring time off versus those who develop chronic stress-related illness. There appears to be more tolerance for acute stress reactions following major incidents, but far less tolerance for long-term cumulative stress issues. Martin reported that officers continue to work in a culture that messages “suck it up and move on” (p. 83). Aside from fear of being perceived as weak by colleagues, officers did not come forward for help when they were clearly struggling for fear of being transferred, having their service pistol taken away, or loosing promotional opportunities. Martin maintained that overall change would occur only with a fundamental transformation in cultural perceptions pertaining to psychological injuries.

Heffren and Hausdorf [20] studied police officers coping with traumatic stress in terms of their exposure, perception and initial help-seeking responses. Based on a sample of 421 web-based surveys distributed in Southwestern Ontario, Canada, they found that comfort levels with personal disclosures and a supportive work environment were critical for officer disclosure. Trusted sources such as family and friends were more frequently sought than professional counsellors. The authors suggested that reframing mental health services as consultations, coaching and workshops might counteract stigma attached to accessing services. They also highlighted the potential for basic stress management training at the initial stage of an officer’s career.

In the fifth study, Karaffa and Koch [40] examined the responses of 248 police officers from Oklahoma and Texas to a 62-item online survey designed to capture attitudes toward mental health and help seeking. The authors found the presence of public stigma and self-stigma predictive of the officers’ decisions to access mental health services. They identified that respondents reporting lower self-stigma scores were more likely to have sought assistance with mental health concerns in the past. Those with the belief that the public had a negative attitude toward mental health concerns experienced higher levels of self-stigma and were less receptive to help seeking. Some of the recommendations to combat their concerns included providing information and education to normalize symptoms and reduce stigma, and increased openness about help seeking from supervisors and respected peers.

Carleton and colleagues [19] in their longitudinal assessment of the R2MR added open-ended questions at the end of their questionnaire and reported that “most participants reported improved understanding of mental health, stronger skills for discussing mental health, improved clarity regarding mental health resources, lower stigma and appreciation of the R2MR tools, particularly the Mental Health Continuum model” (p. 522).

Although the research is growing, there continues to be limited understanding in the police literature related to help seeking. The literature reviewed identified that stigma continues to act as a barrier to help seeking. To our knowledge, there is no known published research exploring factors that influence the decision to seek psychological support in a police population using the ECIT. Therefore, the present study was designed to explore the experiences of RCMP members in order to extend our understanding about factors that help and hinder their decisions to access psychological services. Further, in our study, we identified barriers to help seeking, and offer information about how to minimize barriers and better support police personnel.

## 2. The Method: An Enhanced Critical Incident Technique Study

The method chosen was the Enhanced Critical Incident Technique (ECIT) [41]. With roots in the Critical Incident Technique (CIT) method, developed by James Flanagan [42] during the second world war, the method was initially utilized to conduct job analysis and identify tools to measure job performance. A critical incident is described as an observable behaviour to meet the specific criteria for data collection; the action has to be complete enough to allow an observer to make predictions about the person (criteria for “incident”); and the actual purpose and act has to be clear enough to understand the outcome (criteria for “critical”).

Gremler [43] synthesized 141 CIT studies in the area of service research to determine the strengths and weaknesses of the method. The author concluded that the CIT was an effective tool to collect both information and context from the perspective of the patient. The Enhanced Critical Incident Technique [41] is an extension of Flanagan’s original CIT method and is currently recognized as a reliable and valid method for the human and social sciences and has provided a foundation for research that continues today [41,44]. Butterfield and colleagues [41] adapted the method and named their modified version the *Enhanced Critical Incident Technique*. The authors added a list of nine credibility checks to the method. They also incorporated contextual questions at the beginning of each interview to provide background to the ECIT questions. Additionally, Butterfield and colleagues added a question regarding participants’ wishlist items during the interview. The authors recognized participants as experts in their lived experience. They also encouraged the careful development and use of an interview guide to assist in tracking the emergence of new incidents and categories. A review of studies using the ECIT with police populations supported the use of the ECIT as highly appropriate for this study [27,45]. Given the pragmatic approach within the ECIT, results have been written up in such a way as to inform policy, clinical practice, individual officers and supervisors within the policing culture, and potentially other related fields. The rigorous nature of the ECIT with its nine credibility and trustworthiness checks increases the confidence in the results and ensures the findings accurately represents the participants’ lived experience.

### 2.1. Methodological Procedures

The research question addressed was: What helps and hinders the decision to access psychological services in a police population? In addition to the ECIT which produces a list of substantiated categories, a content analysis was conducted to identify any commonalities and differences among the three main domains of helping, hindering and wishlist items.

### 2.2. Description of the Sample

In all, 20 actively serving RCMP officers, 12 men and eight women, were selected to participate in this study. Participant ages ranged from 27 to 58 years, with a mean age of 44.2 for men and a mean age of 42.5 for women. Years of service ranged from 4 to 36 years, with 17.7 years mean years of service. All participants selected met the inclusion criteria with the exception that the group of participants was entirely Caucasian and therefore did not reflect the cultural diversity of the population of the RCMP in British Columbia. As a limitation of this study, we make recommendations for further research to seek out a more representative sample.

### 2.3. Inclusion Criteria

- Currently serving members of the RCMP in the lower mainland district of British Columbia: All participants were actively posted in a detachment or specialized unit in the lower mainland district.
- Had accessed or considered accessing psychological support from a trained professional. All participants described events across their service in which they considered accessing psychological services.
- Willingness to talk about factors that influenced the decision to access (or not) psychological services from a trained professional across their service. All participants appeared fully engaged during the interviews and were willing to share their experiences and perceptions.

### 2.4. Additional Inclusion Considerations

- Every effort was made to recruit numbers of men and women that were reflective of the gender of serving members currently working in the RCMP. The target sample population was 70% men and 30% women. We amended the participant group to include greater representation of women’s experiences, resulting in a gender composition of 60% (12) men and 40% (8) women in this study.
- RCMP officers from diverse cultural backgrounds: As mentioned, a diversity criterion was not met. Participants responding during the recruitment phase were Caucasian.
- Efforts to recruit individuals with varied experience and service to capture as broad a range of experiences and perspectives as possible: Participants represented rank levels from constable through to staff sergeant.

### 2.5. Exclusion Criteria

- Former clients from the first author’s counselling practice were excluded from participating in this study to prevent dual relationships and any potential influence or power differential.
- RCMP officers who were designated as off duty sick were similarly excluded from participating. While likely able to provide extremely relevant insight, they were viewed as more vulnerable given their status. To minimize risk of harm, the decision was made to interview only actively serving members.

### 2.6. Participant Recruitment

Under the guidance and direction of a senior lower mainland district officer, a recruitment poster was disseminated as an attachment through the RCMP internal e-mail system to all members working in the lower mainland district. Given the sensitivity of the research topic and the barriers identified in the empirical literature, care was taken with the wording of the poster to also encourage participation from members not comfortable accessing psychological support. The response to the recruitment poster was immediate with several respondents expressing their support and desire to participate in what they believed was an important topic.

This study was approved by the University of British Columbia’s Behavioural Research Ethics Board, certificate number H13-00304 and prior to meeting with participants to conduct the interviews, interested candidates were contacted and provided with an overview and general information about the types of experiences they would be asked to recall and discuss. Once their questions or concerns were addressed, all the potential participants expressed an interest in moving forward and an interview was scheduled at a minimum of three days from the initial telephone conversation. We established this process to provide time for the participants to reflect on their experiences, identify incidents they felt were important and meaningful to the research topic, and mentally prepare for participation in the interview. Confidentiality and privacy concerns were discussed at length during the recruitment stage, interview process, and participant member checking procedures to ensure complete transparency and to ensure participants were fully informed. A waitlist was compiled of additional potential participants and they were advised that they would be contacted at a future date if further recruitment was needed. Before the research interview, all participants were provided with informed consent, all agreed to voluntarily participate and signed the informed consent form. This study proceeded with the initial 20 participants identified, all of whom remained committed and fully engaged throughout the process. All participants had a minimum of four years policing experience.

### 2.7. Procedures for Data Collection

Most participants preferred to have the interview conducted in a private room or office at their workplace. The interviews ranging from 57 to 142 min (mean of 85 min) were digitally audio recorded and augmented with field notes written during and immediately following interviews. The ECIT allows participants to determine the importance of incidents, and interview questions are utilized to assist with focus and recall of individual incidents and expansion of details to assist the participants’ access to the meaning being made. The questions were piloted with two RCMP officers who were not involved in this study. The opening questions for the research interview were:

(a) Think back across your service to a time (work related or not) when you considered accessing psychological services. (Participant described incident.)
(b) Did you access any psychological assistance? (Confirmation if the participant accessed psychological assistance.)
(c) What influenced your decision to access psychological support?
(d) What specific factors helped you make this decision?
(e) What specific factors hindered this decision?

If the participant did not seek psychological assistance, we asked:

(f) What influenced your decision not to access psychological support?
(g) What specific factors helped you make this decision?
(h) What specific factors hindered this decision?

In addition to exploring incidents that were helpful or hindering to accessing psychological services, participants were asked to identify anything they wished could have been there for them that may have made a difference. An additional question, referred to as a *wish list* item [41], is incorporated into the ECIT procedure to explore what participants believe would have helped in the situation. The process was repeated until the participants were unable to recall any further significant incidents. A new procedure of the ECIT involves the paraphrasing of key points back to participants following their recall of all helpful, hindering and wish list items to cue the participants’ memory to other factors they might wish to add. This process also serves as an additional participant check ensuring key factors are correctly understood. Paraphrasing of key helpful, hindering and wish list items occurred near the end of the interview, when all incidents had been recalled and participants had nothing further to contribute. We followed this step to ensure that the researcher accurately understood what participants related. In some instances, the participants clarified a point or added additional detail thought to be relevant. As a final step, it was an effective means of summarizing content and added more detail. At the conclusion of the interview, all participants were provided with contact information for psychologists in the lower mainland taken from the RCMP provider list and encouraged to seek assistance from a psychologist to assist with any issues that may have surfaced during the interview process.

### 2.8. Procedures for Data Analysis

In total, 14 of the 20 digitally recorded interviews were transcribed verbatim, and targeted transcription was utilized for the remaining six. Incorporating the process of targeted transcription allowed additional interviews to be conducted beyond the saturation point, adding to the richness of the data and increasing confidence in the results. The recordings were simultaneously reviewed and compared with the transcripts to ensure accuracy.

There are three analytic steps in the method described here:

1. Analyzing the data. A three-step process was used to analyze the large amount of information collected. According to Flanagan [42], the researcher must summarize and organize the information into a useable format, “increasing the usefulness while sacrificing as little of the comprehensiveness, specificity and validity of the information” (p. 344). The three steps include:
  - Frame of reference: Using the general aim and specifications developed as a guide, all incidents are reviewed and those that meet the criteria for a critical incident are included in the results.
  - Category Formation: This is an inductive process. Often starting with the first three interviews, the researcher sorts through the identified incidents, and places them into tentative categories. A general description of each category is written down. Subsequent incidents are then placed into these tentative categories. Categories may need to be redefined and new categories developed as the process evolves. Once all of the incidents have been placed within a category, larger categories may be subdivided into subcategories.
  - General behaviour: Relating to the general aim and intended use of the data, the level of generality and specificity in reporting the data is determined.
2. Interpreting and reporting the data. This is the stage in which errors are more likely to occur, as the process is subjective and relies upon the judgement of the researcher [42]. Flanagan cautions those using his method to ensure clarity and carefully review the process used to collect, analyze, and categorize the data.

### 2.9. Reliability and Validity Procedures

According to Butterfield and colleagues [41], the ECIT identifies nine credibility and trustworthiness checks, all of which were completed.

1. Independent extraction of the incidents. To ensure incidents were identified correctly, two individuals with training and experience using the ECIT independently extracted 25% of incidents from transcripts. We identified five new incidents and the transfer of eight incidents into different categories, which occurred after discussion and 100% agreement between the principal investigator and the independent ECIT examiners.
2. Participant cross checking (interpretive validity). Critical incidents extracted from participant interviews were reviewed by participants to ensure their information had been correctly understood. A document for every participant was created containing all incidents extracted from their transcript, organized into helpful, hindering and wish list items. Respective documents were sent to 10 participants voluntarily agreeing to participate in the validity check along with the tentative categories their incidents had been placed into. A second document containing category definitions was also provided. Participants were asked to review both documents and provide feedback. Participants had the opportunity to add, change or delete any incidents and comment regarding their impressions of the categories. Each participant indicated that they felt the incident extraction sheets were accurate and advised that they did not have any additions, deletions or changes. In addition, each participant indicated that they found the categories made sense and either had personal experience with each category, or found the category to be plausible based on their knowledge and experience with the RCMP. A 100% agreement rate lends significant credibility to the strength and composition of the categories.
3. Two independent judges trained in the ECIT randomly placed 25% of incidents into tentatively formed categories (reliability checks) to ensure the category formation was as accurate as possible. The process resulted in a 98% agreement rate.
4. The point of exhaustiveness (redundancy) at which new categories no longer emerge from the data was tracked (reliability and validity check). Redundancy was reached by the 14th interview. While the additional six interviews added richness and depth, they did not result in any new category formation.
5. Two experts in the area of study reviewed the categories to ensure they were logical and in keeping with what is currently understood about the phenomenon (face validity). Two registered psychologists with extensive experience working with potentially traumatic events, workplace engagement, and organizational stress within the RCMP reviewed the categories. Both advised that they found the research to be highly relevant and important, and that the categories were congruent with their knowledge and experience in serving police.
6. To develop a category, a minimum 25% participation rate is required. With the ECIT, any information falling below that threshold was carefully considered and was included in the results if it appeared to have relevance to the phenomenon being explored. Although one helping category “*Understanding about Mental Health and the Psychological Response to Police Work”* had only a 20% participant rate, it was included as it offered valuable information regarding the importance of educating members and provided juxtaposition to the associated hindering category “*Lack of Understanding about Mental Health and the Psychological Response to Police Work”* and wish list category “*Education on Mental Health and the Psychological Response to Police Work*.”
7. The results have been examined in light of the *existing relevant scholarly literature*. The findings of this study are well supported by the empirical literature, as detailed in the discussion.
8. Accuracy of account. To ensure the incidents were captured and categorized accurately, the exact words of participants were used, and participant member checks were conducted.
9. An individual *trained and experienced in using the ECIT* reviewed interviews numbered 1, 5, 10, 13, and 16 to ensure compliance with the ECIT (interview fidelity).

The reviews were conducted on an ongoing basis, shortly after each interview, to allow incorporation of any suggestions to improve interview style. Initial comments pertaining to the clarity of the research question was offered after the first interview. Feedback on subsequent interviews was extremely positive. Upon completion of the construction of the three main domains—helping, hindering and wishlist categories—a content analysis was conducted, searching for commonalities and differences within the three main category domains. Five main themes were found and will be presented next.

## 3. Results

It is standard practice when utilizing the ECIT to report the helping, hindering and wish list items separately. From the 20 recorded interviews conducted, a total of 676 incidents were extracted. Participants identified 264 incidents that were subsequently categorized as helpful, as the incidents according to participants contained aspects/factors the participants regarded as being helpful in their respective decisions regarding accessing psychological services. In terms of hindering incidents, 258 incidents were described by participants as unhelpful and even detrimental in some cases, when deliberating on whether to access psychological services. In keeping with the ECIT, participants provided 154 wishlist items.

All 676 incidents were sorted into 32 categories: 14 that were helpful (see Table 1), 13 considered hindering (see Table 2), and 5 wishlist topics (see Table 3). To effectively report the interconnections and greater meaning of the findings, the results were organized thematically after conducting a content analysis and are discussed within the context of the following five overarching topics: *systemic factors*, *information and education*, *quality of relationships*, *individual characteristics*, *and organizational processes*.

### 3.1. Systemic Factors

It was readily apparent that the environment in which participants worked strongly influenced their decision to access psychological assistance. A primary factor relates to the culture of policing. Participants in our study described the need to appear mentally and emotionally in control and pressure to conform. Many described an expectation within the culture, and within society, for police officers to be strong yet kind, tough yet compassionate, able to respond appropriately to every emergency, withstanding any pressure or challenge they are faced with. Some participants talked about the fact that it was not acceptable to show any weakness. *“The environment I was in at work was very much of a suck it up attitude”*,—*‘suck it up buttercup attitude’—we are supposed to have thick skin”*. Given the critical and potential life-threatening calls they respond to, and their reliance on colleagues to provide back up, it was considered essential that members remain competent and capable. *“It’s not like you’re installing tiles some days it’s life or death and you depend on other people for that lifeline”*. It was also imperative their team members would know that they could count on them to be there for them, *“You want people to feel that they can rely on you and if they can’t*, *you’re really a useless part of that team”*.

An expectation of emotional control at all times, while helpful in the moment as it allows officers to respond to the matter at hand, was identified by several participants as extremely hindering. Discomfort at revealing emotion to colleagues prevented discussion about accessing psychological care.

*One time I really felt that I could have talked to someone was when a co-worker committed suicide, but I didn’t. Even at the time when it all happened, I was at work that day, and I worked with a bunch of guys and they were very blasé about it and I didn’t feel comfortable to show any emotion so you know I just kind of stayed in the back room and kept doing my job.*

As junior members, some participants described looking to more senior officers to determine their response, working to suppress their emotion until they too became either more desensitized or better able to conceal their emotional reactions. Cultural pressure coupled with a lack of understanding about the importance of addressing their emotions dissuaded some from seeking help. One participant described his conditioning process, beginning with his first confrontation with death following a fatal motor vehicle crash as a junior member:

I remember looking at that (deceased mutilated body) and I go “oh my God, like their life was out of them”; it was just really surreal and it just didn’t seemed to bother anyone except me. And so you start learning quickly you have to take care of business and you cannot show that emotion because that’s weak. Because you’re supposed to be strong, you’re supposed to take care of everything and our job’s not to be weak.

Participants described factors related to stigma and self-stigma, identifying the need to preserve and protect their professional reputations, and that seeking psychological help was suggestive to others of weakness or not having what it takes and thereby potentially damaging their reputation.

*There was never in my time as a cadet and all the way up the mere mention of a psychologist or a shrink had a negative connotation to it. It meant that you weren’t strong enough to do the job; that the pressures of the job were causing you to cripple and that you couldn’t do it on your own. That showed a sign of weakness that perhaps this job is too much for you.*

Some participants expressed concern that accessing psychological services would result in tangible negative consequences including loss of promotion. They described experiences in which they were not selected for positions due to a supervisor’s perception that they were weak. Others described overhearing conversations in which colleagues were denigrated for accessing services. The fear of being blacklisted, or denied opportunities, had a strong silencing effect for many.

*It is a career stopper (seeing a psychologist). I wouldn’t go and talk to someone. So even now I understand there is no stigma, but I am still asking okay, well, what goes back? I recognize it could be detrimental to your career.*

At times, it prevented or delayed access to psychological services, and for several participants who did access services, caused considerable stress and worry they would be discovered, resulting in isolating and secretive behaviour. Some participants expressed grave concern that help seeking would result in being deemed unfit for duty:

Who is going to know? Are they going to find out? How is this going to affect my job? Am I going to come in to work one day and my supervisor is going to say, “I got a phone call from Health Services saying that you’re no longer fit for duty.”

A change in perception and attitude toward mental health and resilience was noted with positive result. *“I think within our organization now, I think we’re doing a better job in being mindful of people’s psyche and how they’re feeling and I think it’s not quite as taboo as it used to be.”* Several participants demonstrated awareness of the potential impact of their work and the importance of self-care. *“We have the inside view of the seediness and grotesque parts of life. We have to maintain our mental health.”* Further, for one participant who was recovering from an injury on the job, *“I look at it (psychological care) as my overall rehab and if my mind isn’t necessarily where it needs to be, my body isn’t necessarily going to follow it as well.”*

While acknowledging there is a long way to go, the change is being noticed. Some participants now equate seeking the assistance of a psychologist with accessing a doctor or dentist, just part of one’s overall wellness, a sign the perception of stigma is diminishing for some.

*I know from some of the things that I’ve seen here, it’s just like going to your dentist or just like going to your doctor, go get a check-up, where they are going to see their person for routine maintenance for whatever they’re dealing with…they come to work, they’re productive at work. What we do as a job the regular general public usually don’t get exposed to the stuff we do. But I think it falls under when you go get your teeth cleaned or medical check-ups—get your emotional check-up.*

Participants were hopeful that the change would continue. In addition to slow but perceptible changes to the police culture, some differences in the way participants described their environment and the impact of their work was evident during the interviews, and not just among junior members. There was awareness by most of the participants of the importance of maintaining good mental health. One participant, who is also a supervisor, spent much of her career working in an environment where members did not talk about their feelings or reactions and remarked how imperative being open and talking about mental health is to changing the culture:

That’s the driving force behind my conversations with people now. You have to talk about it. You have to talk to psychologists and feel okay about doing that. Because my whole service, that’s not what I was brought up with so to speak.

### 3.2. Information and Education

Participants who possessed some knowledge of the psychological impacts of police work were better able to recognize and evaluate their own reactions, understand the importance of being proactive and were more informed about when they should seek psychological assistance. *“I didn’t feel right and knew the signs”*, and *“Okay there are steps that I can take to help deal with this and I’m going to take the steps because I want to make the situation better*.” Participants were more likely to employ proactive coping strategies, seek out the assistance of a psychologist or request a CISD intervention. They were also more likely to view their reactions as a consequence of their employment, rather than a personal weakness or failing, increasing the likelihood they would respond proactively: *“If you want to get this far*, *you have to take care of your mental health or you are not going to make it*. *And that is what I do*.”

Participants who were unaware of the potential risks and psychological impact of police work were less likely to employ strategies that would mitigate the impact of the work, less likely to talk about their experience, or recognize the importance of addressing the impact. “*I was experiencing very strong emotions*. *I probably should have sought psychological help*, *but I never thought of it*, *which surprises me now*.”

Participants expressed uncertainty about the origin or seriousness of the symptoms they were experiencing and were similarly uncertain as to if or when they might seek psychological support.

*Because it wasn’t normal and big things happened when I was on the Watch, I never asked for help because I didn’t really think of it as an option or think that it was necessary at the time. No one threw it out there to me so… I think it wasn’t something I even considered.*

Many did not recognize the importance of dealing with adverse reactions: *“It is not really affecting other people*, *so I don’t know*, *is this as big an issue because it is really only affecting me*.” Participants repeatedly described not accessing psychological services because they did not know that they should or did not recognize the symptoms. *“I had been in a fairly bad stretch for such a long time that it almost felt normal*.”

Working in an environment in which strong emphasis is placed on being perceived as competent and steadfastly reliable conflicts with the notion of help seeking. Participants described that not having adequate knowledge about mental health and related issues meant they were not aware of the importance of getting professional help.

Some participants had never experienced any symptoms, and advised if they had, they did not have a “clue” what to do about it. *“I was new on the unit and didn’t know the resources available*.*” “At three years of service I did not know the services available to help*.” Others expressed uncertainty about when they should access assistance, or they were unsure how bad things had to get, leading to a delay in accessing psychological services or postponing access indefinitely.

*It still stops me because the thing right now, is it a big enough issue that I need to see somebody or will this go away in the next few months…? I don’t know, but then when do you get to the point of saying, maybe I should?*

Participants recognized the importance of help seeking and identified a need for education and awareness within their units. They spoke of the importance of promoting access to psychological assistance as needed and being informed about the detrimental effect of not reaching out.

Participants also expressed a wish for more openness and discussion about the benefits of proactive use of psychologists. One mentioned the need for positive role models and the importance of those in higher ranks openly promoting services of psychologists.

*A well-known sports psychologist spoke on a two-day training course, likening high performance sports teams to members on our unit. If someone humbly would say I am performing at this very high level within the organization, but I recognize part of that success is that I can do what I do because I invite services. This is another tool in your toolbox that you can use to help you perform. It would affect many people in a positive way to hear that.*

### 3.3. Quality and Influence of Relationships

For participants in this study, primary importance was placed on having a trusted and respected relationship, as often safe relationships were instrumental in assisting participants to access psychological services. The importance of trusted relationships cannot be overstated, as this speaks directly to the heart of acceptance of psychological services by members in a somewhat rigid culture defined by conformity to traditions and values that appear in conflict with the notion of help seeking.

*My partner and I were the same age, the same level of service; I think we were both hit pretty hard by it. And his response, if he had of gone “absolutely not, that’s crazy” I don’t know what my reaction would have been; it probably would have changed my perspective. But the fact that we both thought, you know, maybe that’s not such a bad idea. … made us go.*

A significant finding emerging from this study was that 75% of participants (*n*=15) stated that without the intervention of a trusted and respected individual, they would not have received much needed psychological services when they did. Some were completely unaware of their emotional state until a concerned, valued and trusted individual brought this matter to their attention. Others were aware they were not coping effectively, but the idea of seeking psychological support did not enter their mind until it was suggested by a caring other.

*At no point did I think “oh I should go and see a psychologist” until I talked to my girlfriend and I just couldn’t keep it together anymore. I was crying at work and just a basket case. Not until I was really desperately in need of help and support other than from my family or friends. It was only because my girlfriend said you know you should go see this guy that I went.*

The level of persuasion from individuals with influence ranged from gentle suggestion to very blunt and direct approaches *“He (supervisor) put his hands on both sides of my face*, *looked me right in the eye and said ‘Go talk to someone’ and I went*.”

Participants identified the importance of having supportive supervisors as they set the example, offering a template on how members should care for their mental health following difficult events whether work or personal in nature. Participants with supportive supervisors described them as caring, genuine, proactive, confidential, and responsive in the moment when they noticed member(s) struggling.

*He was a good supervisor, a ‘member’s member’ that I didn’t worry about, I trusted him. I was aware of the potential risk to my career, but I trusted him. He talked to me and told me that he didn’t know half a dozen people who have had that experience. I never worried he saw me as deficient. Afterward he did regular ‘check ins’ with me in a thoughtful manner. We had good discussions because of who he was. The night it happened he defused the team well. He took his role quite seriously.*

Supportive supervisors tended to set up interventions, demonstrated concern and awareness of what their subordinates were experiencing and encouraged them to seek psychological services as a matter of course, without fear of repercussion or stigma. Participants with supportive supervisors found they had a profound and lasting imprint and, in several cases, inspired the type of supervisor they became.

*When he did that, he cared enough to do it he recognized it and if you care and recognize and speak about it, although at that moment those were the only words I heard, he had great influence over me. Like my supervisor was to me, I want to be that supervisor to them.*

Unsupportive supervisors were seen to have considerable power and influence on how mental health issues were handled in their unit. Supervisors were often described as “blind” to the struggles experienced by their members, they were openly critical of members, breached confidentiality, were known to make negative and derisive comments about subordinates, guaranteeing most members on their unit made every effort to conceal any mental difficulties or contemplation about accessing assistance.

*Not just the information, the fact that I’m going there at all. One of the officers will criticize people for being off with the flu so to you know for him to find out that anybody’s going to see a counsellor, I’d never disclose that to him.*

Some participants elected to forego psychological assistance out of fear that their supervisors would find out and possibly harm their career. *“…my supervisor spoke to people about what was going on with me*.” Further, *“a second supervisor pulled my file and made the decision about whether or not it was something someone should be affected by and then made selections (staffing) based on his opinion.”*

Some supervisors were not willfully unsupportive or obstructive, but rather lacked the appropriate knowledge or ability to assist members experiencing a personal crisis. According to one participant, *“supervisors are the gatekeepers—you become a victim of the ignorance of your boss which is exactly what happened to me*. *But I didn’t have enough service to go: ‘You’re a goof’*.”

In addition to the importance of trusted family, friends and supervisors, participants’ decisions to access psychological assistance were strongly influenced by co-worker attitudes and role modelling, effective peer support, concerned health care professionals, and experiences in critical incident stress debriefings (CISDs).

Feedback from participants on the use of CISDs was mixed. Most participants found them to be a valuable intervention as long as they were conducted at the proper time by a skilled facilitator, and the group was comprised of appropriate individuals.

*(CISD) is hugely important. It adds so much of a human aspect to an otherwise robotic job that we have the debriefs that we have are so important because it allows people to feel…in a closed environment that’s why our CISDs are closed and confidential and quiet. We all want to care; we all want to feel but it also makes us vulnerable. And we’re not supposed to be vulnerable.*

In addition to adding a “human” component to an environment placing high priority on emotional control and suppression, participants found that CISDs provided an introduction to psychological assistance, normalized reactions, and served to remind them of the potential symptoms they may experience. CISDs also provided an opportunity to interact with a mental health professional, an opportunity that may prove useful with decision making regarding accessing a psychologist in the future.

*I had a critical debrief that I initiated as a supervisor as a result of a fatal crash that one of my constables saw. That was initiated not because I felt I needed the assistance but I could see that he did and that it…it’s just sort of a non-judgmental way of allowing people to open up a bit and decide if they need to access more assistance or not.*

Those who found CISDs unhelpful or hindering cited concerns relating to trust and safety in the group. Some participants felt unsafe or judged by certain individuals present in the debriefing, and/or having other inappropriate people in the room.

*We had a debrief shortly after (bloody multiple homicide scene) and the psychologist said:“Anything that you’d like to add?” “Nope” “Really?” “Nothing” and I was the dude inside. Goes to my partner, “You like to say anything?” “Nope”. Because the OIC (Officer in Charge) was sitting there. So, based on that experience there was no way I was going to talk to anybody.*

Participants also expressed concerns regarding timing, sporadic implementation, and the decision about who to include. “There was a debriefing without us. I was a main member involved. I found out after the fact that I was not invited to the CISD. It made me feel resentful.” Several negative experiences were attributed to improper participant selection, as well as what was assessed as an inappropriate facilitator.

*She made really inappropriate comments. I didn’t relate to her, felt awkward. It was like she was talking to me like a five year old kid whose parents were divorcing. Made me feel those psychologists were all the same. She had no idea. How could she understand?*

CISDs were identified by participants as one gateway into the mental health stream. An inappropriate facilitator not only stifled participation in the debriefing but for some participants, caused them to delay or reject accessing psychological services all together. *“I would have talked to someone sooner if I did not have the CISD*, *due to the “flaky” psychologist.”*

Experiences with psychologists had similar trajectories for officers. Participants with positive experiences with a psychologist found them to be genuine and trustworthy and tended to utilize those services as part of a proactive strategy to maintain mental wellbeing. They were more likely to seek assistance and encourage colleagues in need to do the same. *“Having a relationship with a psychologist becomes a tool to call upon*. *He has a good grasp on police life*, *police culture.”* Another stated: *“I have gone back twice for check-ups if I am not doing okay*. *She understands the career perspective*, *and also the emotion—she can work with the whole picture.”*

Those who had a negative experience(s) described lengthy delay or outright refusal to seek subsequent psychological services.

*I don’t think she (psychologist) knew anything about police work. She didn’t come across to me as empathetic or caring enough. The office setting was cold, there was minimal eye contact, she wrote seven pages, I counted them as she flipped while I was talking. And said nothing reassuring, knew nothing about the work. I actually felt worse when I left than when I went and I thought, “What did she write for seven pages about me?” And I think when I left there unconsciously, I thought: “I’ll never see another psychologist again if that’s what it’s about”. And I never went to another psychologist again.*

These experiences speak to the powerful influence of early and subsequent experiences on the decision to seek psychological services, and the need to ensure the provision of quality supervision and culturally informed psychological support.

### 3.4. Individual Characteristics

Possessing self-awareness and personal wisdom were attributes identified by participants as extremely helpful in making the decision to access psychological support. Participants with the ability to recognize the problem on their own and a strong desire to make a change were more likely to access psychological services. Some participants also described having the ability to take action, recognizing that if they wanted things to be different, they would require help. *“I recognized it wouldn’t go away on its own*, *so I called a friend for the number to a psychologist”*, *“I just recognized the stressors*. *And I thought*, *you know what*, *I need to go*. *And I went.”*

Other participants described life experience and witnessing colleagues suffer as motivating to their decision to seek psychological help.

*Experiences, life experience, seeing too many people not deal with it, not deal with their issues and seeing what the consequences are. Causing that self-reflection to go, “I don’t ever want to get there; I don’t ever want to be one of those so how do I avoid it?”*

Further, for several participants with service and seniority, it was the decision not to pursue further promotions that made the difference.

*I said okay enough is enough, the soldiering on isn’t working. I knew it wasn’t working and frankly I’m at a point in my career where even if it does, if that belief that it’s going to hurt me, it’s not going to hurt me and I’m probably maxed out, I don’t anticipate trying for another promotion.*

Being exposed to messages valuing strength, giving, helping others, and dealing with life challenges alone certainly hinders the decision to seek psychological assistance. Participants who were raised in an environment that reinforced emotional constraint and the message that “helpers do not seek help” expressed difficulty with the concept of accessing psychological services for themselves. This delayed or prevented access to psychological services. “*It’s not appropriate; it’s the vulnerableness of it that I don’t like*. *My role is always to help the vulnerable not be the vulnerable person*.”

This attitude and belief pushed some of the participants to carry on despite the difficulties they were experiencing, until, for some, the pressure built to the point that it became overwhelming and they were forced to accept help. Further, even with that experience of receiving help, the power of this negative perception lingered.

*Would I see a psychologist? I have. Since I’ve got this new position I don’t anymore. I think it would be a pretty tragic thing for me to have to go and see someone. I’d have to have that melt down again to go and talk to someone.*

Unique and interesting findings emerging from our study relate to help seeking and gender differences. As the results show, 87% of women participants identified the police culture as hindering in contrast to 33% of men; and 75% of women participants delayed access to psychological services until their emotional state had deteriorated to the point that they were no longer able to cope emotionally in contrast with 8% of men. The remaining participants recognized they were in need of assistance much earlier, or utilized services proactively.

Participant responses revealed two vital points pertaining to the frequency of help seeking and gender differences. First, it is abundantly clear that many of the participants in this study did not access psychological assistance as frequently as they felt they should, and in fact, did not access psychological services for a considerable percentage of work-related incidents—53% for women and 44% for men—often due to cultural expectations.

*It’s a very masculine environment that we work in and it’s one of those things where you don’t want to be lesser than anybody else, you want to be an equal, you want to be that sort of macho type personality where “hey look, I’m okay, I can go and do all of this work” and you go and do the work and you can’t really think that it’s not affecting anybody with some of the stuff that we see in the unit. It’s not talked about.*

Secondly, women officers interviewed for this study were more likely to find the police culture hindering, delaying access to psychological services until they were in significant distress, and overall, less likely than the men to seek psychological help. Some women participants stated that to be viewed as equal, they felt they had to prove themselves to a greater degree than the men at work.

*Number one they allowed us (women) to come in to the Force, number two dear God if you have a woman who’s weak and crying and seeing a psychologist it’s even worse. Why did you allow them to come into this Force? So I think there’s a huge difference in the sexes based on that. For me to be a woman number one, I am not as physically strong and if I ever overtly said I was going to see a psychologist it would make it even worse, because they’d say why did they ever allow us in? I have heard those comments many times over my service. So that probably has some bearing. I have to be strong because I have to show - I may be a woman, but I can still cut the mustard!*

### 3.5. Organizational Processes

To promote resilience and healthy attitudes toward help seeking in a population that is exposed to early and ongoing potentially traumatic events and organizational stressors, it is important to ensure the policies and practices promote an environment of strong psychosocial safety and proactive response. Some participant experiences involved moments of inspired leadership, promotion of mental wellbeing, and effective support. “*I think the organization is doing a pretty good job now*. *Not only identifying*, *but having the resources there*. *I haven’t seen anybody or even myself not get something that I asked for*.”

Participants identified a number of helpful initiatives including access to a list of psychologists with experience working with police officers.

*I was able to choose from anyone on that list. And on the list it actually says what they deal with so critical incident, law enforcement, that kind of thing, or maybe if they deal mostly with relationship issues… so it actually delved into the particular portion of psychology that they deal with which helped me narrow down my field of search. It was enormously helpful; instead of just going ennie mennie miney moe…I could actually make a semi-informed decision on which I could potentially go to.*

The Performance Assessment Review system is designed to track the frequency of a member’s attendance at potentially traumatic incidents. It flags individuals who attend multiple difficult files so that supervisors can check in on them. As with many processes, this was perceived as helpful or potentially helpful depending on the effectiveness of the supervisor responsible for the follow up.

*One of the things they do now is they’ve got this automatic system and there’s certain things that occur within your day to day life that are reported on. If an accumulative total is reached then it automatically triggers a review and then they say to the manager okay this person maybe needs to go see somebody or you need to pay attention to him. I think they call it Performance Assessment Review or something like that, PAR. But it depends on who it is that comes to talk to you.*

As with many processes, helpfulness was often directly connected with the participant’s perception of the intention behind the practice or of the individuals implementing it.

*… in these cases they won’t prevent you from accessing counselling, they may force you to go to counselling, but then they’ll use it against you. So that’s the problem with the system. The process is a good idea, it’s the human element that is unpredictable and can make the process an adverse part of the member’s life.*

Participants described their involvement in several major incidents as very junior members with no guidance or instruction on how to deal with the emotional impact. For some, it felt like there was no one to care, no one to notice when they were not doing well. This, at times, created a barrier to help seeking, as the process to ask for help became too overwhelming.

*I think when you’re handed something and you’ve got a physical piece of paper that says “this is what you can do and this is important for you to do, this is what other people do” that all of a sudden it seems like a more acceptable practice. But when it’s like okay this is available to you and it’s way over here and you’ll have to go through all these series of passwords on the Internet to go access them or whatever it is or try to find them on the mutual shared drive, maybe you can find it, maybe not but you might have to ask someone else where to find it. But you don’t want to. It’s really not something at the top of a priority list. It becomes too overwhelming.*

There were several organizational factors identified as helpful and hindering to help seeking. In addition to police organizations providing education, information, appropriate policy and procedures, creating a climate in which members are able to feel supported in being proactive about their mental health relies upon *consistent and effective implementation of policies and procedures*, including acceptance and engagement of this process at all levels and by all individuals within the police organization.

The following six key points emerged from the theme *organizational process*.

#### 3.5.1. Importance of Promoting and Encouraging All Employees to Look Out for Colleagues and Assist Those Who May Be Struggling

The critical role of trusted and respected individuals to assist members recognize changes to their mental state and help them to access a psychologist that they could trust and feel comfortable with is vital. This requires informed colleagues and supervisors prepared to have courageous conversations about a potentially difficult and sensitive topic and willingness to remain involved and offer follow up as required.

#### 3.5.2. Use of Senior Members as Role Models

The data also highlights the opportunity for greater influence when credible senior members offer proactive support, information, and positive role modeling regarding the importance of mental wellbeing and accessing psychological services to junior members, particularly when the senior members are highly respected.

#### 3.5.3. Critical Incident Stress Debriefings.

Implementing *CISDs* after major incidents using trained, trusted psychologists with knowledge of police culture in a timely manner with all individuals involved (as appropriate). Supervisors must be educated so that they can accurately assess the need, support their use and encourage attendance. Those unable or unwilling to attend the CISD who had a key role in the incident should be encouraged to check in with a psychologist as a matter of routine to prevent any member from falling through the cracks.

#### 3.5.4. Mandatory Psychological Check Ins

Given the well-established fact that most members are exposed to ongoing potentially traumatic events and organizational stress, and that traditional values of the police culture conflict with help seeking, it is recommended that mandatory annual psychological check ins are implemented for all employees. This demonstrates the organization’s commitment to and the importance of mental wellbeing, provides a routine check in, and presents an opportunity for members to establish a relationship with a care provider, increasing the likelihood they will access future psychological services if needed.

#### 3.5.5. Police-Informed Psychologists

Police orgnizations need to ensure the psychologists on the provider list are knowledgeable about police culture and aware of some of the key barriers to help seeking with this population. The data suggests that members will be more likely to access psychological services from professionals they know, trust, and have a previous relationship with. Efforts to expose members to the psychologists available to them would be beneficial. Consideration should be given to involving psychologists in periodic ride a longs, watch briefings, training days and CISDs.

#### 3.5.6. Early Exposure to Attitudes Regarding the Potential Impact of the Work and Mental Health Care

High priority should be placed on focusing attention and resources on the education and promotion of knowledge about the impact of police work, assisting members in understanding the range of reactions, resources available and steps they can take to proactively care for their mental wellbeing. Mental health literacy is key to changing stigma and discrimination in police culture.

## 4. Discussion

The decision to access psychological services was often guided by the level of knowledge and understanding participants held about the potential psychological impacts of police work and the attitudes of those around them. Those who had greater awareness were more likely to employ proactive strategies and seek psychological interventions, while those with little understanding or support were more likely to perceive their reaction as a form of weakness, internalize and isolate themselves—some electing to cope instead with increased alcohol consumption and other unhealthy coping strategies, a finding well supported in the literature [46,47].

It is remarkable to note that so many of the participants in this study believed they would not have received help were it not for the intervention of caring, trusted others. This finding is also supported in the recent empirical literature [14,20,27]. This emphasizes the importance of social support and should provide incentives to police personnel and others to reach out to officers who may be unaware they are at risk. Findings suggest individuals who are well connected to officers who are struggling can have an instrumental role in facilitating their decision to seek psychological support, as without their intervention, assistance may not be accessed.

While all relationships were influential, of note were officers’ experiences with supervisors and culturally informed psychologists. Carleton and colleagues [14,19] also emphasized the importance of appropriate and trustworthy key personnel in police leadership. Supervisors and senior officers provided role modelling for junior members about how to deal with emotional and potentially traumatizing incidents. Participants who had supervisors who were genuine and proactive in their response to major incidents, checking in with them, normalizing reactions, arranging CISDs and encouraging them to seek psychological assistance when thought appropriate, were more likely to develop a similar framework from which to respond to subsequent traumatic events. This template later influenced their actions when they became supervisors themselves. Those with unsupportive, uninvolved, or uninformed supervisors were more likely to experience difficulty and embarrassment accessing psychological assistance. Some accessed psychological services surreptitiously, although their access was often delayed. Several participants described learning from those difficult experiences, choosing to adopt a more supportive approach when they became supervisors themselves.

Experiences with psychologists had similar trajectories for officers. Those with positive experiences, connecting with a psychologist that they found genuine and trustworthy, utilized those services as part of a proactive strategy to maintain mental wellbeing. They were more likely to seek assistance and encourage colleagues in need to do the same. Those who had a negative experience(s) described lengthy delay or outright refusal to seek subsequent psychological services.

The culturally valued practice of emotional control also emerged as a major barrier, delaying or preventing access to psychological services. While often necessary and beneficial in the moment during a police response, emotional suppression is identified in the empirical literature as potentially harmful to overall psychological wellbeing [6,8,25]. In this study, it hindered the decision to access psychological services. Several participants described extreme discomfort with their emotion, fearing that “opening the box”, particularly after many years of suppressing intense emotions, would result in an inability to maintain control, and thus be perceived as less competent and reliable at work. Individuals too concerned or fearful to discuss the negative impacts of their work deny themselves access to guidance that they might otherwise receive from experienced colleagues who could play a critical role in directing officers to psychological services.

The predominant role of relationships in influencing the decision to seek psychological services emerging from this study reinforces the importance of encouraging the establishment of strong social and professional connections designed to promote acceptance, support and encouragement for officers to take steps required to maintain their mental wellbeing [48,49]. The findings speak to the issue of safety and ensuring officers feel secure and comfortable in their environment and with those around them, as this too guides their decision-making processes to seek help.

Some unique and important findings emerging from this study relate to the frequency of help seeking and gender. Empirical literature on men who seek help identified a lower incidence of help-seeking behaviour among men [5,7]. Those findings were contradicted in this study, with women participants slightly less likely than their male counterparts to seek psychological assistance for work related issues. Given the congruence between masculine characteristics and those of the “ideal” police officer, it is theorized that in a bid for acceptance in this male-dominated culture, some women officers may adopt hyper-masculine traits [25,50,51]. Carleton and colleagues [9,14] discuss this issue and propose that further research is needed to understand gender differences in help seeking among police personnel. We agree and extend this call for future research to include examination of other diversity factors that influence help-seeking decisions among police.

An additional unique contribution to the theoretical literature reveals differences between frequencies of access to psychological services for work events versus personal events. In this study, men and women participants accessed the services of a psychologist 89% of the time for personal events such as marital difficulties, divorce, death of child or family member; and only 53% of the time for work events. The findings offer unique and important information that would benefit from future study.

### Conclusions

A career in policing requires officers to be both mentally and physically fit, ready to respond to any request for service they are dispatched to. As our knowledge about the causes and treatment of mental health issues increases, including the impact of police work, our understanding about strategies to prevent or mitigate harm also develops. Focusing efforts on prevention, education and timely treatment for those exposed to potentially traumatic events and the numerous stressors of police work can assist members and employees to develop resilience and remain strong and healthy in their work.

In addition to ensuring appropriate processes and education are in place, a consistent message from participants in this study was the importance of relationships. Police cultural values promote emotional suppression and objectivity, while help seeking requires a level of vulnerability [52,53]. The likelihood messaging will be effective and members more inclined to reach out for help when needed is often connected to the level of faith and trust they have in the people around them. R2MR education will likely be more meaningful when delivered by respected personnel; CISDs will be experienced as more beneficial and supportive when facilitated by well trained, experienced and culturally aware psychologists with the appropriate people in the room; supervisors who are perceived as genuinely caring will have a greater impact when checking in with their team members. Members of police organizations, colleagues, families, friends and professionals, including mental health professionals, have critical roles to play in the creation of these psychologically safe environments.

Although there has been previous qualitative research on the topic of help-seeking behaviour among police populations [54], this is the first study exploring factors that help and hinder the decision to access psychological services in a police population. A number of the findings are supported by existing empirical literature, increasing the validity of this study. In addition, our research findings extend extant knowledge and offer specific information about the many inter-related facets associated with help seeking in police populations. The results of this study add to and are relevant to the work being conducted by Carleton and colleagues [55] on the RCMP longitudinal PTSD study at the University of Regina. Our findings support the need for further research on help-seeking decision making among police.

Based on the experiences of actively serving RCMP members working in the lower mainland division in British Columbia, our study draws from examples of successful and unsuccessful contacts, interventions, and processes that either helped or hindered the decision to access psychological services. The findings may serve to inform current serving members and employees regarding help seeking and offer insight and suggestions to supervisors and managers to assist them in their efforts to support employees and promote mental health and wellbeing. The pragmatic information includes several detailed suggestions and recommendations intended to facilitate a supportive psychosocial environment that promotes mental wellbeing (see Table 4). As police organizations continue with their efforts to enhance resilience and develop and maintain healthy operational workplaces, organizational leaders now have additional information upon which to plan future programming, and base their decisions and policies within evidence-based research.

## 5. Limitations of the Research

Participants represented a wide range in age, years of service, and rank. However, there were no officers from an ethnic minority background involved in this study. It is important to note that the results reflect the experiences and opinions of Caucasian men and women RCMP officers only. As it is crucial to develop an understanding of factors that help and hinder the decision to seek psychological services for all officers, it would be beneficial to conduct similar studies targeting specific populations to ensure inclusion of the experiences of all officers.

Given the limited number of participants interviewed for this study (*n* = 20), the results reflect the experiences of the participants only, and while serving to inform the larger police and related organizations, they cannot be generalized. As such, the findings are offered tentatively.

Our study was conducted with currently serving RCMP members in the lower mainland of British Columbia, and while some participants also had experience working in rural postings, the majority of the data came from their experiences in the metro Vancouver area. There are significant differences between policing in rural and urban centers including resources, funding, access to services, and the manner of policing that is practiced. As the RCMP is a national police force, there are also differences between each division (province/territory) and type of duties, including federal and provincial units with a variety of responsibilities. While some of the information contained within this study may resonate with members in rural postings, in other areas and jurisdictions across Canada, and in other police forces, it is important to specify that data is primarily reflective of RCMP participants working in the context of the lower mainland of British Columbia.

Findings from a limited number of qualitative studies have previously addressed the stigma and potentially traumatic experiences of police. Although this study replicates other qualitative research, it is unique in that it was the first study to use the ECIT to understand factors that help or hinder help-seeking decision making among the RCMP in a targeted geographical area—the lower mainland of British Columbia.

## Figures and Tables

**Table 1:** Incidents That Helped the Decision to Access Psychological Services.

Category of Incident	Number of Incidents (Percentage of Total) *264 Helping Incidents*	Number of Participants (Percentage of Total) *N = 20*
1. Influential Third Party	39 (15%)	18 (90%)
2. Ability to Talk about Life Circumstances, Self-Awareness, and Desire to Change	36 (14%)	17 (85%)
3. Psychologist	30 (11%)	16 (80%)
4. Threshold for Accessing Psychological Services	25 (9%)	15 (75%)
5. Ease of Access to Psychologist	24 (9%)	13 (65%)
6. Supportive Unit and Supervisor	24 (9%)	10 (50%)
7. Greater Awareness/Acceptance of Mental Health Issues, Changing Culture	16 (6%)	10 (50%)
8. RCMP Organizational Processes	13 (5%)	10 (50%)
9. Critical Incident Stress Debriefing (Personal Experience)	13 (5%)	7 (35%)
10. Previous Experience with Counselling	10 (4%)	7 (35%)
11. Knowledge of Resources	8 (3%)	7 (35%)
12. Mandatory Psychological Interventions	12 (3%)	6 (30%)
13. Member/Employee Assistance Program (Personal Experience)	8 (3%)	5 (25%)
14. Understanding Mental Health and the Psychological Response to Police Work	4 (2%)	4 (20%)

**Table 2:** Incidents That Hinder the Decision to Access Psychological Services.

Category of Incident	Number of Incidents (Percentage of Total) 258 Hindering	Number of Participants (Percentage of Total) *N* = 20
1. RCMP Culture	43 (17%)	15 (75%)
2. Lack of Understanding about Mental Health and the Psychological Response to Police Work	26 (10%)	13 (65%)
3. Unsupportive Supervisors/Coworkers	24 (9%)	12 (60%)
4. Stigma Re: Help Seeking	26 (10%)	11 (55%)
5. Lack of Knowledge of Services Available/Entitled To	22 (9%)	11 (55%)
6. Fear of Repercussion	19 (7%)	10 (50%)
7. Critical Incident Stress Debriefing (Personal Experience)	15 (6%)	10 (50%)
8. Member/Employee Assistance Program (Personal Experience)	12 (5%)	9 (45%)
9. Perceived Lack of Support or Care for Mental Wellbeing	17 (7%)	8 (40%)
10. RCMP Organizational Processes	15 (6%)	8 (40%)
11. Psychologist	7 (3%)	6 (30%)
12. Upbringing: Family Messages, Personal Characteristics	9 (3%)	5 (25%)
13. 1-800# (Personal Experience/Opinion)	5 (2%)	5 (25%)

**Table 3:** Wish List Items.

Category of Incident	Number of Items (Percentage of Total) *154 wish list*	Number of Participants (Percentage of Total) *N* = 20
1. Organizational Processes	39 (25%)	19 (95%)
2. Promoting Psycho-Social Care and Implementation of Critical Incident Stress Management (CISM) Procedures	38 (25%)	17 (85%)
3. Information on Services/Entitlements	26 (17%)	12 (60%)
4. Effective Supervisors	18 (12%)	12 (60%)
5. Education on Mental Health and the Psychological Response to Police Work	29 (19%)	10 (50%)

**Table 4:** Recommendations for Police Organizations.

1. Provide education to all members and their families on services and entitlements. This should occur prior to a member experiencing a crisis and continue periodically throughout a member’s career. Suggestions include commencing at depot, at first posting, ongoing at block training and related courses.
2. Clearly post information on services and service providers in all detachments and/or units so members are able to access information quickly and with little effort.
3. Develop information packages for recruits and following transfers to new detachments and units. Include all policy related to psychological wellness, resources specific to the geographical area, and self-care information, including links to websites.
4. Supply a copy of a regularly updated psychological provider list to all members, with a notation that members are not strictly confined to the service provider list.
5. Provide exposure and training to psychologists on the nature and demands of police work and the police culture.
6. Educate members about the psychological services available to them (how can it assist them, what to expect when they make contact, and issues related to confidentiality).
7. Ensure individuals providing in-house support (peer to peer) are carefully selected, highly trained and solely or primarily dedicated to these duties.
8. Update information websites to ensure medical and psychological resources are readily accessible.
9. Develop effective messaging and communication regarding services available, particularly during times when major incidents have occurred or were noted. Establish a process so that members who are off duty as a result of a psychological or physical injury or are involved in an internal or public complaint are proactively reminded of resources available to them.
10. Supervisors: Ensure training is provided to supervisors on the impact of police work (primary and secondary trauma, impact of cumulative exposure, and organizational stress), symptoms to look for in their subordinates, the importance of promoting mental health and psychological care as a necessary aspect of the job. Ensure supervisors are knowledgeable about all policy related to psychological health and wellness. Develop a system to ensure supervisors are accountable to policy, particularly regarding implementation of trauma interventions and mandatory annual assessments.
11. Implement mandatory psychological “check ins” (i.e., safeguard program) for all members and employees working for police organizations.

Note: Since the interviews were conducted, the RCMP implemented Road to Mental Readiness (R2MR) training nationally. Originally developed by the Department of National Defense, the program focuses on providing information and education related to mental health, reducing stigma and accessing care [11,12,16].
